# Enhancement of biological properties in casein protein through functionalization with cinnamaldehyde *via* Schiff base bonding: antibacterial and antioxidant effects

**DOI:** 10.1039/d5ra00852b

**Published:** 2025-07-31

**Authors:** Aisha Hendy, Nazly Hassan, Jehan El-Nady, Amal S. I. Ahmed, Rabab Mohamed Abou Shahba, Tamer M. Tamer

**Affiliations:** a Chemistry Department, Faculty of Science (Girls), Al-Azhar University Nasr City Cairo Egypt; b Composites and Nanostructured Materials Research Department, Advanced Technologies and New Materials Research Institute (ATNMRI), City of Scientific Research and Technological Applications (SRTA-City) New Borg El-Arab City, P.O. Box: 21934 Alexandria Egypt; c Electronic Materials Department, Advanced Technologies and New Materials Research Institute (ATNMRI), City of Scientific Research and Technological Applications (SRTA-City) New Borg El-Arab, P.O. Box: 21934 Alexandria Egypt; d Polymer Materials Research Department, Advanced Technologies and New Materials Research Institute,(ATNMRI), City of Scientific Research and Technological Applications (SRTA-City) New Borg El-Arab City, P.O. Box: 21934 Alexandria Egypt ttamer85@gmail.com

## Abstract

This study investigates the modification of casein protein with cinnamaldehyde *via* Schiff base bonding, aiming to enhance its biological properties. The functionalization process was validated through different analytical techniques, including Fourier Transform Infrared Spectroscopy (FTIR), which confirmed the formation of Schiff base linkages. Thermal Gravimetric Analysis (TGA) and Scanning Electron Microscopy (SEM) were employed to assess the thermal stability and morphological changes of modified protein, respectively. Biological evaluations revealed that the cinnamaldehyde-functionalized casein exhibited significant antibacterial activity against four different bacteria strains [two Gram-positive (*Staphylococcus aureus* and *Bacillus cereus*) and two Gram-negative (*Escherichia coli* and *Pseudomonas aeruginosa*)], showcasing its potential as an antimicrobial agent. Antioxidant tests demonstrated enhanced scavenging abilities, suggesting protective effects against oxidative stress. Furthermore, preliminary hemocompatibility assessments indicated that the modified protein is non-toxic to human blood cells, underscoring its suitability for biomedical applications. Collectively, these findings suggest that cinnamaldehyde-functionalized casein holds promise for advancing healthcare materials.

## Abstract 2

This study investigates the modification of casein protein with cinnamaldehyde *via* Schiff base bonding, aiming to enhance its biological properties.

## Graphical abstract

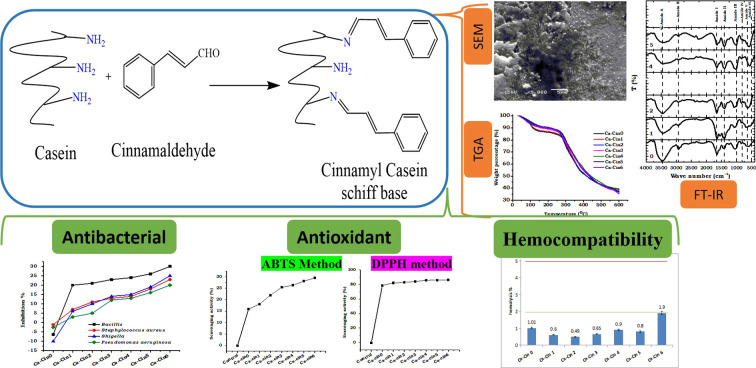

## 1. Introduction

Casein, the predominant phosphoprotein found in milk, exhibits unique properties that make it a valuable material in various sectors, including food, pharmaceuticals, and biomedicine.^1^ Casein was formulated of multi-essential amino acids, including glycine, leucine, tyrosine, and arginine.^2^ Despite its intrinsic biocompatibility and functional versatility, the practical applications of casein are often limited by its moderate performance in critical areas such as microbial resistance and oxidative stability. Consequently, enhancing these properties could significantly expand casein's utility, especially in biomedical applications where antimicrobial and antioxidant characteristics are crucial.^3^

**Fig. 1:**
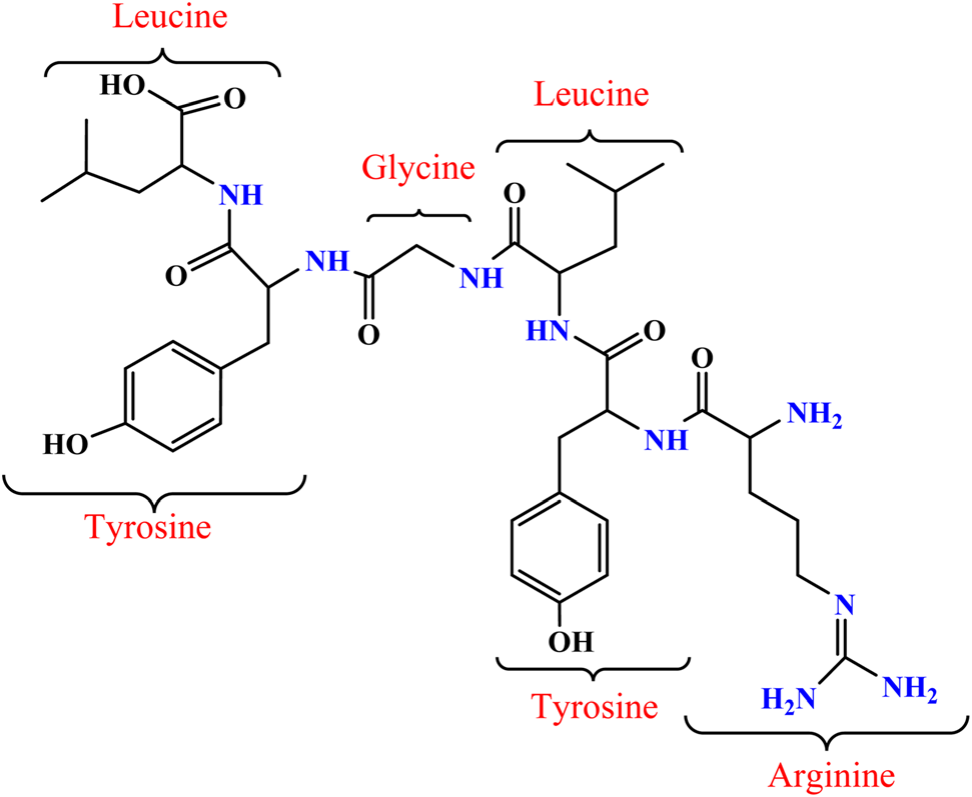
Representative structure of casein protein highlighting key amino acid residues with functional groups (*e.g.*, –NH_2_) that are suitable for Schiff base conjugation with cinnamaldehyde.

The chemical functionalization of proteins (as presented in Fig. 1) has emerged as a valuable strategy for enhancing their biological and physicochemical properties, making them more suitable for a range of advanced applications, especially in biomedicine and materials science.^4^ Among the various modification techniques, Schiff base formation, achieved by the reaction of primary amine groups in proteins with aldehydes, stands out for its efficiency and versatility. This approach is particularly advantageous because Schiff base bonds—characterized by the forming imine linkages—are both reversible and exhibit notable stability under physiological conditions, which is critical for maintaining functionality in biological environments. The stability and ease of formation of Schiff bases make them a popular choice for protein modification, offering new avenues to optimize protein performance for targeted applications.

Recent advancements in protein modification have highlighted the potential of Schiff base formation to enhance protein properties for biomedical applications. Gelatin, a widely used protein, has been frequently modified *via* Schiff base linkages with aldehydes to improve its bioactivity and physicochemical stability. For instance, El-Meligy *et al.* (2022) successfully prepared gelatin–aldehyde derivatives, demonstrating that the Schiff base modification not only enhanced the structural integrity of gelatin but also improved its thermal and mechanical properties, which are essential for biomedical use.^5^ Similarly, Hassan *et al.* (2024) explored the functionalization of gelatin with cinnamaldehyde as a natural aldehyde with known antimicrobial activity to create a bioactive gelatin derivative.^6^ This modification showed significant potential in combating multidrug-resistant bacteria, as confirmed by *in vitro* antimicrobial assessments and supported by molecular docking and pharmacokinetic analyses. These studies underscore the versatility of Schiff base formation in tailoring the properties of gelatin for applications where enhanced stability and bioactivity are crucial, paving the way for innovative materials in wound healing and antimicrobial therapies.

Cinnamaldehyde, the organic compound responsible for the characteristic flavor and aroma of cinnamon, is highly regarded for its antimicrobial and antioxidant properties,^7^ rendering it a promising candidate for the modification of biopolymers *via* Schiff base reactions.^8^ The functionalization of biopolymers such as casein, chitosan, and gelatin with cinnamaldehyde has been demonstrated to significantly enhance their biological properties, including antibacterial and antioxidant capabilities.^9,10^ This enhancement broadens the potential applications of these modified biopolymers in areas such as antimicrobial coatings, wound dressings, and drug delivery systems.^11–14^ Research has highlighted the efficacy of cinnamaldehyde-modified materials, including cinnamyl chitosan Schiff bases, which exhibit robust free radical scavenging properties, and cinnamaldehyde-functionalized gelatin, which shows antibacterial activity against multidrug-resistant bacteria. These advancements underscore the potential of cinnamaldehyde in the development of biomaterials characterized by improved oxidative stability and bioactivity for biomedical applications.

This study aims to explore the biochemical synthesis of cinnamaldehyde-functionalized casein *via* Schiff base bonding and evaluate its biological properties. Comprehensive assessments through Fourier Transform Infrared Spectroscopy (FTIR), Thermo Gravimetric Analysis (TGA), and Scanning Electron Microscopy (SEM) are employed to characterize the structural and thermal properties of the modified protein. Additionally, the modified protein's biological efficacy is assessed *via* detailed antibacterial, antioxidant, and hemocompatibility evaluations, aiming to establish a foundation for future applications of this novel bioconjugate in the medical and pharmaceutical fields.

## 2. Materials and methods

### 2.1. Materials

Casein bovine (Sigma, Germany), cinnamaldehyde 98% (Scharlau, Spain), sodium hydroxide 99% (El-Nasr Pharmaceutical Co. for Chemicals, Egypt). Ethanol (99.9%) and sulfuric acid (98%) are supplied from International Co. for Supp & Med. Industries, Egypt. Yeast extract (Merck, Germany), peptone (Bioshop, Canada Inc.), sodium hydrogen carbonate (99%, Fluka, Germany). Potassium persulfate (K_2_S_2_O_8_) (Merck, Germany), 2,2′-azinobis-[3-ethylbenzothiazoline-6-sulfonic acid]diammonium salt (ABTS ≥ 99%), and 2,2-biphenyl-1-picrylhydrazyl (DPPH) (Fluka, Germany) were used.

### 2.2. Methods

#### 2.2.1. Synthesis of cinnamyl-modified casein Schiff base

To synthesize the casein-based Schiff base derivative, 1.0 g of casein was initially dissolved in 50 mL of 0.1 N sodium hydroxide solution under ambient conditions with constant stirring. Following complete dissolution, 10 mL of ethanol containing predetermined amounts of cinnamaldehyde was gradually introduced into the alkaline protein solution to ensure thorough mixing. The resulting mixture was then subjected to continuous stirring at 50 °C for 3 hours to facilitate Schiff base formation, which was evidenced by the development of a deep yellow coloration. After the reaction was completed, the product was isolated and dried in a vacuum oven maintained at 60 °C overnight. To ensure removal of any residual, unreacted cinnamaldehyde, the dried material was washed several times with ethanol. Six formulations were prepared using varying concentrations of cinnamaldehyde (0.05, 0.1, 0.15, 0.2, 0.25, and 0.3 g). These correspond to casein-to-cinnamaldehyde weight ratios of 1 : 0.05, 1 : 0.10, 1 : 0.15, 1 : 0.20, 1 : 0.25, and 1 : 0.30, respectively. They were designated as Ca-Cin1 through Ca-Cin6, respectively, alongside the unmodified casein sample, referred to as Ca-Cin0.

### 2.3. Characterization of polymer

#### 2.3.1. Physico-chemical properties

##### 2.3.1.1. Determination of ion exchange capacity

To evaluate the ion exchange capacity (IEC) of native casein and its Schiff base derivatives, 0.1 g of each sample was dispersed in 20 mL of 0.1 M sulfuric acid (H_2_SO_4_) solution. The suspensions were left to equilibrate for 2 hours at room temperature to allow sufficient interaction between the sample and the acid medium. Following equilibration, the mixtures were filtered, and the filtrates were subjected to titration using 0.1 M sodium hydroxide (NaOH) solution. A blank titration was carried out in parallel under identical conditions, excluding the addition of casein, to serve as a control. The IEC was quantified by comparing the volume of NaOH required to neutralize the residual acid in the presence and absence of the casein material. The IEC was calculated using the following equation:
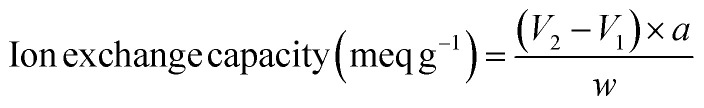
where *V*_2_ represents the volume (in mL) of NaOH used in the control titration, *V*_1_ is the volume used in the sample titration, *a* is the normality of the NaOH solution, and *w* is the mass (in grams) of the casein or derivative sample analyzed.^15^

##### 2.3.1.2. Solubility test

To assess solubility, a measured quantity of the sample was introduced into a buffered solution with varied pH levels and stirred continuously at room temperature for three hours. Afterward, any undissolved solid was separated by filtration, dried, and weighed.^16^ The solubility percentage was then calculated using the formula:
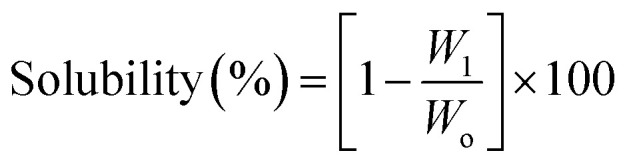
where *W*_1_ represents the weight of the undissolved residue, and *W*_o_ denotes the initial weight of the sample.

##### 2.3.1.3. Moisture content

Samples were placed in humidity chambers maintained at 80% relative humidity and left overnight. Following this, each sample was weighed both before and after drying in an oven at 105 °C for two hours. The moisture content was calculated using the formula:
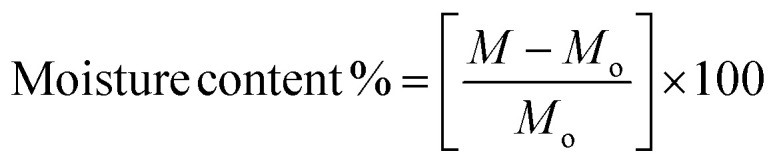
In this equation, *M* denotes the weight of the sample prior to drying, and *M*_o_ is the weight after drying.

#### 2.3.2. Spectral characterization

##### 2.3.2.1. Fourier transform infrared spectroscopy (FTIR)

Infrared (IR) spectra for both casein and casein Schiff base samples were obtained using a Fourier Transform Infrared (FT-IR) spectrometer (Shimadzu FTIR-8400S, Japan). Spectral data were collected over the wavelength range of 4000 to 400 cm^−1^, with each measurement comprising 30 scans at a resolution of 4 cm^−1^.

##### 2.3.2.2. Thermal characterization

The thermal behavior of native casein and its Schiff base derivatives was assessed through thermogravimetric analysis (TGA) using a Shimadzu TGA-50/50H instrument (Japan). Approximately weighed samples were subjected to a programmed heating regime from room temperature to 600 °C at a constant heating rate of 10 °C per minute. The analysis was conducted under an inert nitrogen atmosphere, maintained at a continuous flow rate of 20 mL min^−1^, to prevent oxidative degradation during thermal decomposition.

##### 2.3.2.3. Scanning electron microscopy (SEM)

The surface morphology of casein and its Schiff base derivatives was examined using a scanning electron microscope (JEOL JSM-6360LA, Japan). Prior to imaging, powdered samples were mounted onto aluminum stubs using conductive carbon adhesive. To improve surface conductivity and image resolution, the specimens were sputter-coated with a thin layer of gold under vacuum conditions.

##### 2.3.2.4. Antibacterial evaluation of casein and casein Schiff bases

The antibacterial activity of native casein and its cinnamaldehyde-modified Schiff base derivatives was assessed according to the method described by Wiegand *et al.* (2008),^17^ with minor modifications. Four bacterial strains were selected for the evaluation, including two Gram-positive (*Staphylococcus aureus* and *Bacillus* spp.) and two Gram-negative (*Pseudomonas aeruginosa* and *Shigella* spp.). All strains were initially cultured in Luria–Bertani (LB) broth containing 1% peptone, 0.5% yeast extract, and 1% sodium chloride. The cultures were incubated at 37 °C with shaking at 200 rpm for 24 hours to ensure adequate bacterial growth.

After the initial incubation, 10 μL of each bacterial suspension was inoculated into 10 mL of fresh sterile LB broth supplemented with 0.2 mL of either native casein or one of the casein derivative solutions (2% w/v). These protein solutions had been previously sterilized by autoclaving at 121 °C for 20 minutes. Each treatment group was incubated under the same conditions (37 °C, 200 rpm) for an additional 18 hours.

To ensure the validity of the results, a negative control group was included. This control consisted of bacterial cultures incubated in fresh LB broth without the addition of any casein or derivative compounds, thereby establishing a baseline for bacterial growth in the absence of potential antimicrobial agents.

Following incubation, bacterial growth was quantified by measuring the optical density (OD) at 620 nm using a UV-visible spectrophotometer. The percentage of antibacterial activity was calculated according to the following formula:
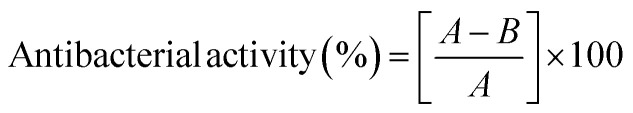
where *A* represents the optical density of the negative control (bacteria without treatment) and *B* represents the optical density of the test sample (bacteria treated with casein or its Schiff base derivatives).

##### 2.3.2.5. Antioxidant evaluation of casein and casein Schiff bases

###### 2.3.2.5.1. DPPH method

The antioxidant potential of native casein and its Schiff base derivatives was assessed using the 2,2-diphenyl-1-picrylhydrazyl (DPPH) radical scavenging method, as described by Olszowy and Dawidowicz (2018),^18^ with slight modifications. A stock solution of each protein sample was prepared at a concentration of 0.1% (w/v) in 0.1 M sodium hydroxide. To initiate the reaction, 100 μL of the protein solution was mixed with 2 mL of a 0.1 mM DPPH solution prepared in 95% ethanol. The mixture was gently shaken and kept in the dark at room temperature for 30 minutes to prevent light-induced degradation.

Following incubation, the absorbance of each reaction mixture was recorded at 517 nm using a UV-visible spectrophotometer. A control sample was prepared under identical conditions using 100 μL of ethanol in place of the protein solution. The degree of DPPH radical scavenging activity was inferred from the reduction in absorbance and calculated using the following formula:
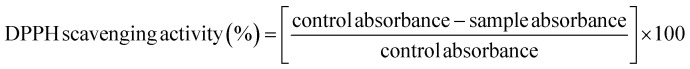


###### 2.3.2.5.2. ABTS method

The radical scavenging ability of casein and its Schiff base derivatives was investigated using a modified ABTS assay, adapted from the method reported by Rapta *et al.* (2009).^19^ The ABTS˙^+^ radical cation was generated by reacting 17.2 mg of ABTS with 3.30 mg of potassium persulfate dissolved in 5 mL of deionized water. This mixture was allowed to stand in the dark at a temperature below 0 °C overnight to ensure complete formation of the stable bluish-green ABTS radical cation.

Before conducting the assay, the resulting ABTS˙^+^ stock solution was diluted with distilled water to a final volume of 60 mL to adjust the absorbance. Protein solutions (0.1%) of both unmodified casein and its derivatives were prepared in 0.1 M sodium hydroxide. For each assay, 100 μL of the sample solution was added to 2 mL of the diluted ABTS˙^+^ solution. The mixtures were vortexed gently and incubated in the dark at room temperature for 30 minutes to allow the scavenging reaction to proceed.

The absorbance of each sample was then recorded at 734 nm using a UV-1800 spectrophotometer (SHIMADZU, Japan). The percentage of ABTS radical scavenging activity was determined using the same formula applied in the DPPH assay:
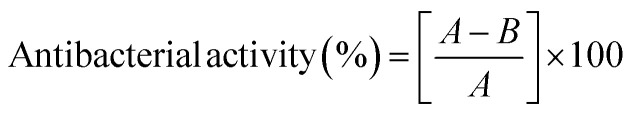
where *A* represents the absorbance of the ABTS˙^+^ solution without protein, and *B* denotes the absorbance in the presence of the test compound.

##### 2.3.2.6. Evaluation of hemocompatibility

The hemolytic potential of casein and its Schiff base derivatives was assessed by measuring the release of hemoglobin from red blood cells upon exposure to the test materials, following a modified protocol based on the method described by Saebo *et al.* (2023).^20^ For each test, 0.1 g of the sample was dissolved in 10 mL of sodium bicarbonate (NaHCO_3_) solution at a concentration of 10 000 ppm. To prepare the erythrocyte suspension, 8 mL of freshly collected anticoagulated human blood was diluted with 10 mL of normal saline (NS).

Each test was conducted by mixing 6 mL of the prepared polymer solution with 4 mL of NS to reach a total volume of 10 mL. Negative controls consisted of 10 mL of NS, while positive controls comprised 10 mL of distilled water to induce complete hemolysis. All samples were pre-incubated in a water bath at 37 °C for 30 minutes prior to the addition of blood. Subsequently, 0.2 mL of the diluted blood sample was introduced into each tube, followed by a second incubation at 37 °C for 60 minutes to facilitate erythrocyte interaction with the materials.

Post-incubation, the mixtures were centrifuged at 2000 rpm for 15 minutes to separate intact cells from the supernatant. The degree of hemolysis was determined by measuring the absorbance of the supernatant at 540 nm using a UV-visible spectrophotometer (Ultrospec 2000). The hemolysis percentage was calculated using the following formula:
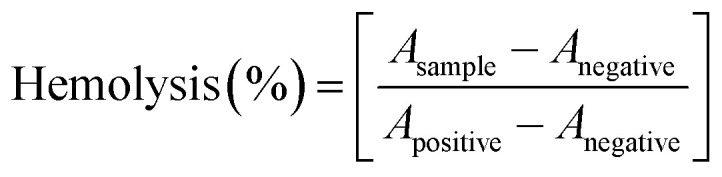
where *A*_sample_, *A*_negative_, and *A*_positive_ represent the absorbance values of the test sample, negative control, and positive control, respectively.

Human blood used in this study was obtained from a healthy adult volunteer in accordance with the standard protocol for testing hemolytic properties of materials (ASTM F756-00), international ethical guidelines, and our previously published studies (Omer *et al.*, 2021;^21^ Tamer *et al.*, 2021 ^22^). All procedures were approved by the SRTA-City-Institutional Ethics Committee, and informed consent was obtained from the donor prior to sample collection.

## 3. Results and discussion

**Fig. 2:**
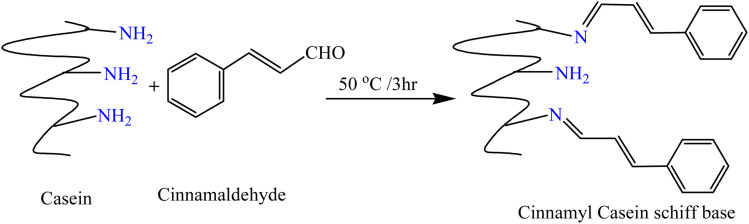
Reaction scheme of casein–cinnamaldehyde Schiff base preparation.

In the present study, a series of casein-based Schiff base derivatives functionalized with cinnamaldehyde were synthesized with the aim of enhancing the intrinsic biological properties of casein. The formation of the Schiff base was facilitated by the condensation reaction between the primary amine groups of casein and the aldehydic carbonyl groups of cinnamaldehyde, leading to the development of stable imine (C=N) linkages, as depicted in Fig. 2. Structural confirmation of the modifications was performed using Fourier-transform infrared spectroscopy (FT-IR), thermogravimetric analysis (TGA), and UV-visible spectroscopy. These techniques verified the successful chemical conjugation and provided detailed information regarding the thermal stability and electronic transitions of the modified derivatives. Furthermore, scanning electron microscopy was employed to investigate the morphological alterations in the casein matrix following functionalization. The subsequent sections present a comprehensive evaluation of the physicochemical characteristics, antioxidant potential, and antibacterial activity of the cinnamaldehyde-modified casein derivatives, underscoring their potential utility as multifunctional bioactive materials.

### 3.1. Physico-chemical properties

The ion exchange capacity (IEC) of the synthesized casein–cinnamaldehyde Schiff base derivatives was determined to evaluate how chemical modification affects the functional characteristics of casein. The IEC values, expressed in milliequivalents per gram (meq g^−1^), were recorded as follows: 2.4 ± 0.12 for unmodified casein (Ca-Cin0), followed by a gradual decline across the modified samples—2.1 ± 0.105 (Ca-Cin1), 1.9 ± 0.095 (Ca-Cin2), 1.7 ± 0.085 (Ca-Cin3), 1.5 ± 0.075 (Ca-Cin4), 1.2 ± 0.06 (Ca-Cin5), and 0.5 ± 0.025 (Ca-Cin6), respectively. This decreasing trend in IEC correlates with increasing degrees of modification and provides evidence of progressive Schiff base formation. The reduction in IEC is attributed to the diminished availability of free amine groups, which are consumed during the formation of imine (C=N) linkages with cinnamaldehyde. These amine groups, when protonated, contribute to ion exchange functionality; thus, their depletion results in fewer protonated ammonium sites (–NH_3_^+^), subsequently lowering the material's ion exchange capacity. These findings confirm the successful chemical conjugation and underscore the significant alteration of casein's functional properties through Schiff base formation, which may influence its applicability in biological and pharmaceutical systems.^23^

The solubility behavior of both unmodified casein and its cinnamaldehyde-functionalized derivatives was also examined across a pH range of 4 to 12 to assess the influence of functionalization on aqueous stability. Native casein displayed solubility in the pH range of 6 to 12, with a marked decrease at acidic pH values, particularly below its isoelectric point (pH 4.6), where protein precipitation occurred. This behavior is characteristic of casein, as the net charge of its amino acid residues is neutral at the isoelectric point, promoting aggregation and precipitation of micelles.^24^ Above this pH, the acquisition of a net negative charge enhances electrostatic repulsion, improving solubility and colloidal stability.

In contrast, the solubility of the Schiff base derivatives was significantly influenced by the extent of cinnamaldehyde substitution. Lower degrees of substitution (Ca-Cin1 to Ca-Cin3) exhibited solubility profiles comparable to native casein between pH 6 and 12. However, higher substitution levels (Ca-Cin4 to Ca-Cin6) led to a pronounced decline in solubility across the entire pH spectrum. This reduction is primarily due to the replacement of hydrophilic amine groups by hydrophobic cinnamyl moieties during the Schiff base formation. As a result, the hydrophilicity of the protein matrix decreases, leading to reduced aqueous solubility. These observations highlight the critical role of molecular modification in tuning the physicochemical behavior of casein and suggest that increased hydrophobicity associated with higher substitution may impact its performance in biomedical and material applications (Table 1).

**Table 1:** Solubility percent of casein and casein Schiff bases at different pH

	Ca-Cin0	Ca-Cin1	Ca-Cin2	Ca-Cin3	Ca-Cin4	Ca-Cin5	Ca-Cin6
pH = 4	0	0	0	0	0	0	0
pH = 6	100	100	100	100	55	31	25
pH = 7	100	100	100	100	74	72	61
pH = 8	100	100	100	100	75	74	73
pH = 9	100	100	100	100	85	83	78
pH = 12	100	100	100	100	98	91	86

The moisture content of native casein and its Schiff base derivatives was analyzed to evaluate the influence of cinnamaldehyde functionalization on the hydrophilicity of the protein matrix. Native casein (Ca-Cin0) exhibited a moisture content of 22.5 ± 1.12%, with a consistent decrease observed among the modified derivatives: 19.1 ± 0.955% (Ca-Cin1), 18.0 ± 0.9% (Ca-Cin2), 15.3 ± 0.765% (Ca-Cin3), 14.0 ± 0.7% (Ca-Cin4), 12.8 ± 0.64% (Ca-Cin5), and 10.0 ± 0.5% (Ca-Cin6). This downward trend indicates a progressive reduction in water retention capacity as the degree of chemical modification increases.

The observed decline in moisture content is attributed to the substitution of hydrophilic primary amine groups with hydrophobic cinnamyl moieties through Schiff base formation. The introduction of these non-polar groups reduces the overall polarity of the protein surface, thereby decreasing its affinity for water molecules. This transformation limits the material's ability to absorb and retain moisture from the surrounding environment, reflecting an increase in hydrophobic character. The correlation between reduced moisture content and higher degrees of substitution provides further confirmation of successful chemical modification and its impact on the physicochemical behavior of casein.

### 3.2. Spectral characterization

#### 3.2.1. Fourier transform infrared spectroscopy (FTIR)

**Fig. 3:**
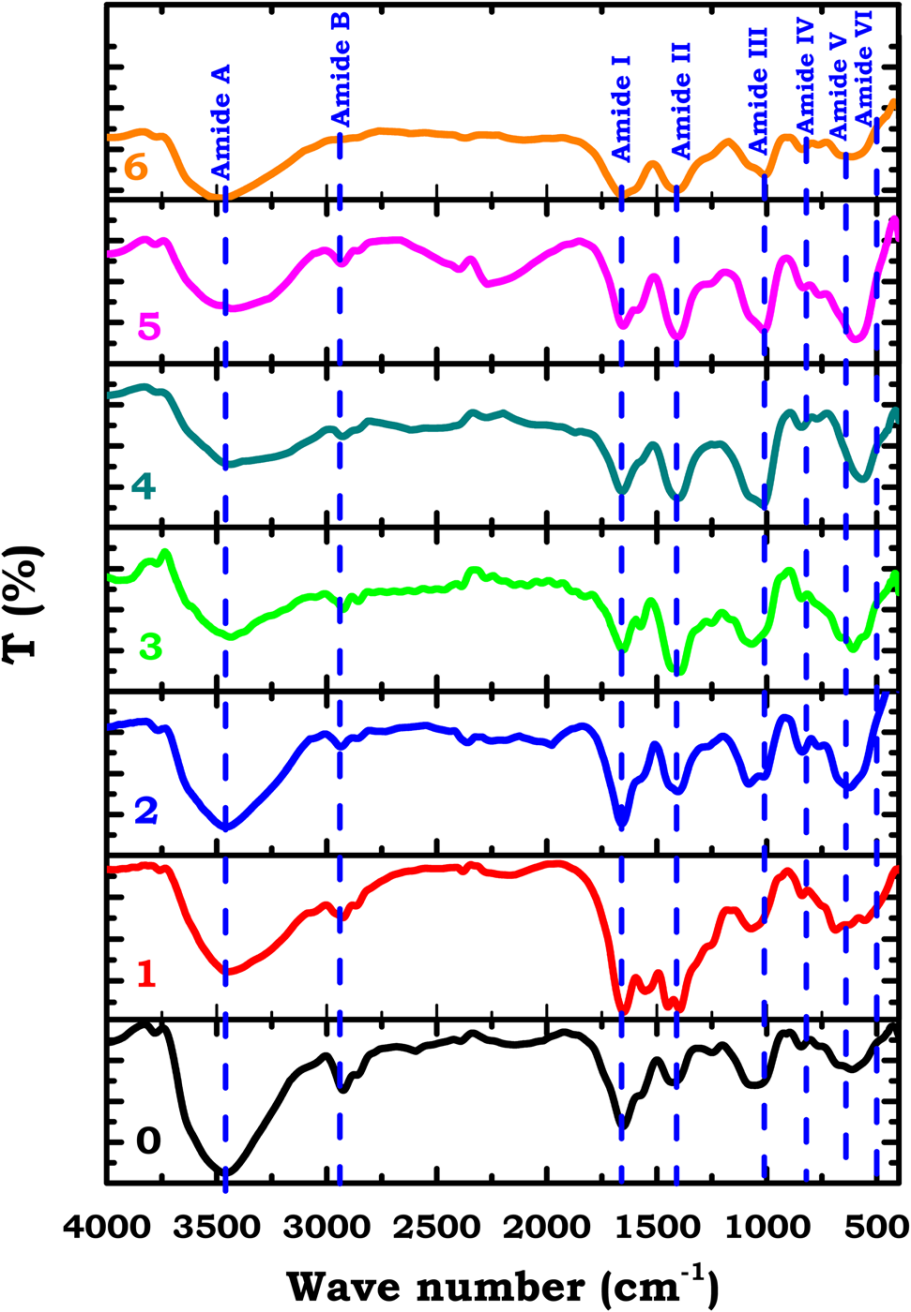
FT-IR of casein and its Schiff base derivatives.

Fourier Transform Infrared (FTIR) spectroscopy was employed to investigate the structural features of native casein and its cinnamaldehyde-functionalized Schiff base derivatives, as depicted in Fig. 3. FTIR is a widely utilized analytical technique for identifying functional groups and assessing molecular modifications, particularly in protein-based systems, due to its sensitivity, speed, and reliability.^25^ The FTIR spectrum of unmodified casein (Fig. 3) displayed characteristic absorption bands attributed to protein amide vibrations. These included the Amide A band (3300–3100 cm^−1^), associated with N–H stretching; Amide B bands (2944 and 2867 cm^−1^) corresponding to C–H stretching; the Amide I region (1600–1690 cm^−1^) indicative of C=O stretching; Amide II bands (1480–1575 cm^−1^) resulting from N–H bending; and Amide III bands (1229–1301 cm^−1^) representing C–N stretching vibrations.^26,27^

Following the functionalization of casein with cinnamaldehyde, the FTIR spectra of the Schiff base derivatives exhibited notable modifications. A new or intensified band appeared around 1620–1640 cm^−1^, which may be attributed to the imine (–C=N) stretching vibration, suggesting Schiff base formation. However, we recognize that this region may also include contributions from residual C=O or C=C groups of unreacted cinnamaldehyde or overlapping amide bands, and thus the assignment should be interpreted with caution. Additionally, new peaks observed in the 1200–1260 cm^−1^ region were associated with phenolic functionalities introduced by the cinnamaldehyde moiety, providing further evidence of chemical coupling.^28^ A decrease in the intensity of the Amide I and Amide II bands was also observed in the modified samples, suggesting the partial consumption of amine groups during the condensation reaction. These spectral changes, including shifts and reduced band intensities, point to alterations in the secondary structure of casein and the incorporation of new functional groups.

#### 3.2.2. Thermal characterization

**Fig. 4:**
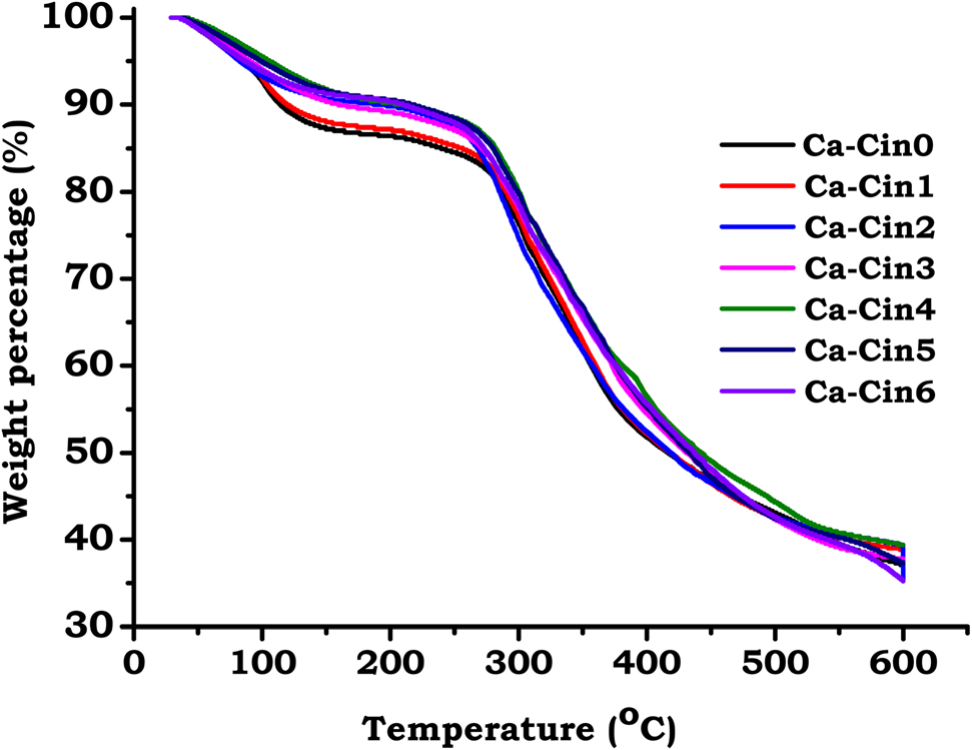
Thermal gravimetric analysis (TGA) of casein and its Schiff base derivatives.

Thermogravimetric analysis (TGA) was conducted to investigate the thermal stability and decomposition behavior of native casein and its cinnamaldehyde-modified Schiff base derivatives, as illustrated in Fig. 4. The TGA profiles for all samples revealed three primary stages of thermal degradation. The first stage, observed between 60 °C and 150 °C, corresponds to the loss of moisture, including both free and bound water. This water loss is attributed to the hydrophilic nature of casein, which contains amino acid residues capable of binding water through physical adsorption and hydrogen bonding.

Quantitative analysis of mass loss in this initial phase revealed a value of 12.2% for unmodified casein (Ca-Cin0), followed by a gradual reduction in the modified samples: 11.3% (Ca-Cin1), 11.57% (Ca-Cin2), 8.79% (Ca-Cin3), 8.6% (Ca-Cin4), 7.5% (Ca-Cin5), and 6.8% (Ca-Cin6). This decreasing trend in moisture content with increasing degrees of substitution indicates a decline in the material's hydrophilic character. The reduction is likely due to the consumption of polar amine groups during Schiff base formation, coupled with the incorporation of hydrophobic cinnamaldehyde moieties. These modifications reduce the protein's ability to interact with water molecules, resulting in lower water retention, as observed in the TGA profiles.

The second stage of thermal decomposition, occurring between approximately 220 °C and 341 °C, is attributed to the pyrolytic breakdown of the protein matrix. This phase, responsible for an estimated 21% of total mass loss, involves the cleavage of peptide chains and the degradation of structural protein components. During this stage, the disruption of disulfide linkages within the casein network is accompanied by the evolution of sulfur-containing gases, such as sulfur dioxide (SO_2_) and hydrogen sulfide (H_2_S).^29^ The introduction of Schiff base linkages appears to influence the degradation pathway, likely through modifications in molecular stability and conformational rigidity induced by cinnamaldehyde functionalization.

The final stage of mass loss, observed within the temperature range of 400 °C to 600 °C, corresponds to the oxidative decomposition of residual carbonaceous material. This exothermic process results in the near-complete oxidation of organic remnants, culminating in further weight reduction. The alterations in thermal behavior across all stages suggest that Schiff base formation not only increases the hydrophobicity of casein but also modulates its thermal stability. These observations support the successful chemical incorporation of cinnamaldehyde into the protein framework and are in agreement with previous studies on thermally analyzed chemically modified proteins.^29^

#### 3.2.3. Morphological characterization

**Fig. 5:**
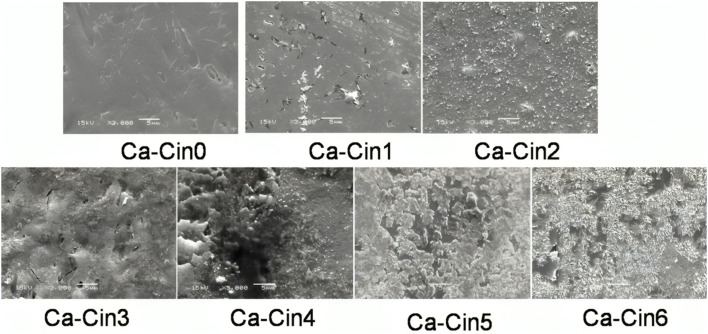
Scanning electron microscope of casein (Ca-Cin0) and six different casein Schiff bases: Ca-Cin1, Ca-Cin2, Ca-Cin3, Ca-Cin4, Ca-Cin5, and Ca-Cin6.

The morphological characteristics of native casein and its cinnamaldehyde-functionalized Schiff base derivatives were examined using Scanning Electron Microscopy (SEM), as illustrated in Fig. 5. The SEM images reveal notable alterations in surface architecture as a result of the chemical modification process. Unmodified casein (Ca-Cin0) displayed a relatively smooth and homogeneous surface, indicative of its native protein structure. In contrast, the Schiff base derivatives exhibited a marked increase in surface heterogeneity and roughness, which became more pronounced with higher levels of cinnamaldehyde substitution (Ca-Cin1 through Ca-Cin6). These progressive morphological changes suggest a structural reorganization of the protein matrix induced by the introduction of cinnamyl groups, likely reflecting the impact of Schiff base formation on the microstructural properties of casein.

The observed increase in surface roughness is likely a consequence of the structural reorganization induced by Schiff base formation, wherein cinnamaldehyde molecules are covalently linked to the casein backbone. This chemical modification introduces bulky hydrophobic cinnamyl groups, which disrupt the native organization of the protein matrix and contribute to the development of a more irregular and textured surface. The incorporation of aromatic aldehyde functionalities has previously been associated with enhanced surface hydrophobicity and increased topographical complexity, as reported in related polymer modification studies.^30,31^ Such structural alterations are characteristic of Schiff base functionalization, where the addition of rigid aromatic moieties modifies the physical architecture of the original biopolymer.^32^

These morphological transformations may offer functional advantages for biomedical applications, particularly in the fields of tissue engineering and scaffold design. Increased surface roughness enhances the available surface area, which can facilitate improved interactions with biological systems. It is well established that topographically complex surfaces promote cellular responses such as adhesion, proliferation, and tissue integration—particularly for dermal fibroblasts—thereby supporting their use in regenerative therapies.^31,32^ Accordingly, the morphological changes observed not only verify the successful conjugation of cinnamaldehyde to the casein matrix but also highlight the potential of these modified biopolymers for use in wound healing and bioengineered scaffolds.

### 3.3. Bioevaluation of the modified casein

#### 3.3.1. Antibacterial evaluation of casein and casein Schiff bases

**Fig. 6:**
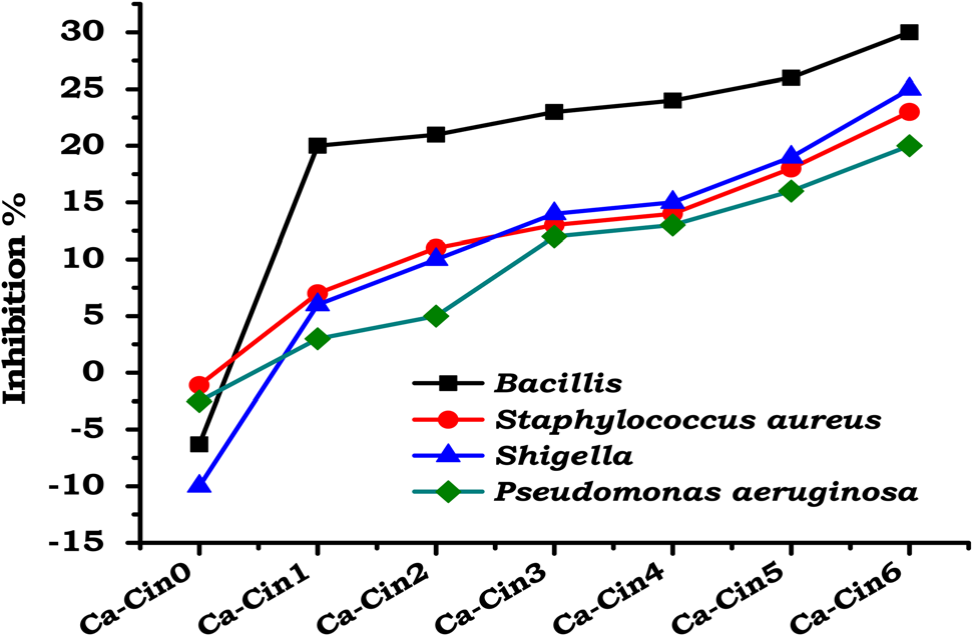
Antibacterial activity of native casein (Ca-Cin0) and cinnamaldehyde-functionalized casein derivatives (Ca-Cin1 to Ca-Cin6) against four bacterial strains: *Staphylococcus aureus*, *Bacillus* spp., *Pseudomonas aeruginosa*, and *Shigella* spp. The experiments were performed in triplicate (*n* = 3). A negative control group (bacterial culture without casein or derivatives) was included to serve as the baseline for assessing antibacterial efficacy.

The antibacterial properties of native casein and its Schiff base derivatives functionalized with cinnamaldehyde were assessed against a panel of bacterial strains, including two Gram-positive species (*Staphylococcus aureus* and *Bacillus*) and two Gram-negative species (*Pseudomonas aeruginosa* and *Shigella*), as shown in Fig. 6. The unmodified casein (Ca-Cin0) exhibited no observable inhibitory effect against *S. aureus* or *P. aeruginosa*, suggesting limited inherent antimicrobial potential. Notably, an apparent stimulatory effect on bacterial proliferation was observed in the case of *Bacillus* and *Shigella*, which may be attributed to the nutritional composition of casein. As a protein rich in amino acids and peptides, native casein can potentially support microbial growth by serving as a supplementary nutrient source.

Following functionalization with cinnamaldehyde, the casein Schiff base derivatives exhibited a moderate enhancement in antibacterial activity. This improvement is likely attributed to the incorporation of phenolic aldehyde moieties, which possess inherent antimicrobial properties. Despite this modification, the overall antibacterial effect remained relatively limited, particularly against Gram-negative strains. This reduced efficacy may be explained by the structural characteristics of Gram-negative bacteria, such as their outer membrane, which serves as an effective barrier against a wide range of antimicrobial agents.^33^

The modest antimicrobial performance of the Schiff base derivatives could also be linked to a low degree of functional substitution and the partial preservation of the native casein's hydrophilic nature. These factors may hinder sufficient hydrophobic interactions between the modified protein and bacterial membranes, thereby limiting disruption of cellular integrity.

These results suggest that although cinnamaldehyde functionalization introduces some antibacterial potential, it is insufficient for achieving broad-spectrum antimicrobial activity. To enhance the efficacy of such materials, further modifications—such as the incorporation of antibiotics or additional antimicrobial agents—may be necessary. This combinatorial approach could yield synergistic effects, potentially improving the therapeutic utility of the modified casein derivatives for biomedical applications.^34^

#### 3.3.2. Antioxidant evaluation of casein and casein Schiff bases

In this study, the antioxidant activity of casein and casein Schiff base derivatives was done using two common methods (ABTS and DPPH).

##### 3.3.2.1. DPPH method

**Fig. 7:**
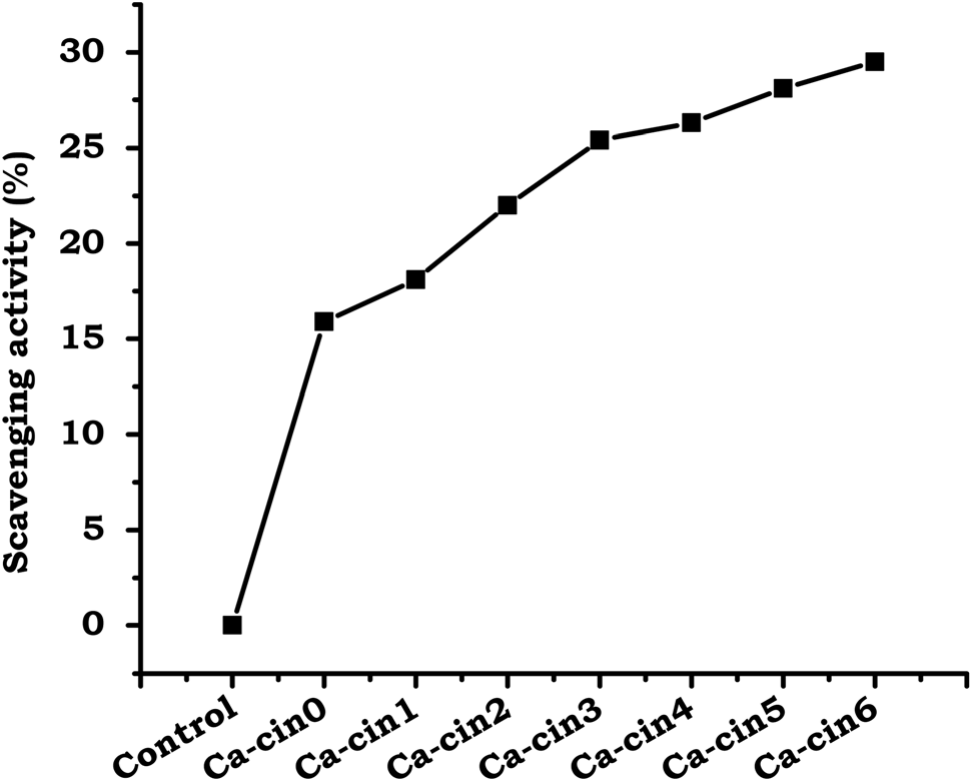
DPPH radical scavenging activity of native casein (Ca-Cin0) and cinnamaldehyde-functionalized casein derivatives (Ca-Cin1 to Ca-Cin6). The results are expressed as the mean ± standard deviation of three independent experiments (*n* = 3).

The antioxidant potential of native casein and its cinnamaldehyde-functionalized Schiff base derivatives was evaluated using the DPPH free radical scavenging assay, as illustrated in Fig. 7. The DPPH assay is a standard method for assessing antioxidant activity by monitoring the reduction of the stable, violet-colored DPPH radical, which turns yellow upon accepting an electron or hydrogen atom from an antioxidant molecule.^35^ The extent of discoloration, measured as a decrease in absorbance at 517 nm, is directly proportional to the radical scavenging capacity of the sample.

Fig. 7 shows that the cinnamaldehyde-modified casein derivative exhibited a more pronounced decrease in DPPH absorbance compared to native casein. This improvement in antioxidant activity is attributed to the electron-donating properties of the cinnamyl groups introduced *via* Schiff base formation. The phenolic structure of these moieties likely donates electrons, thereby facilitating a more efficient neutralization of the DPPH radicals. Consequently, the enhanced radical-quenching ability observed in the modified casein is indicative of the successful incorporation of antioxidant functional groups.

These results corroborate earlier reports that biopolymers functionalized with phenolic compounds display improved antioxidant capacities.^6^ The increased scavenging activity of the cinnamaldehyde-functionalized casein suggests its potential as an effective antioxidant agent for applications in food preservation, pharmaceutical development, and biomedical formulations aimed at mitigating oxidative stress.

##### 3.3.2.2. ABTS method

**Fig. 8:**
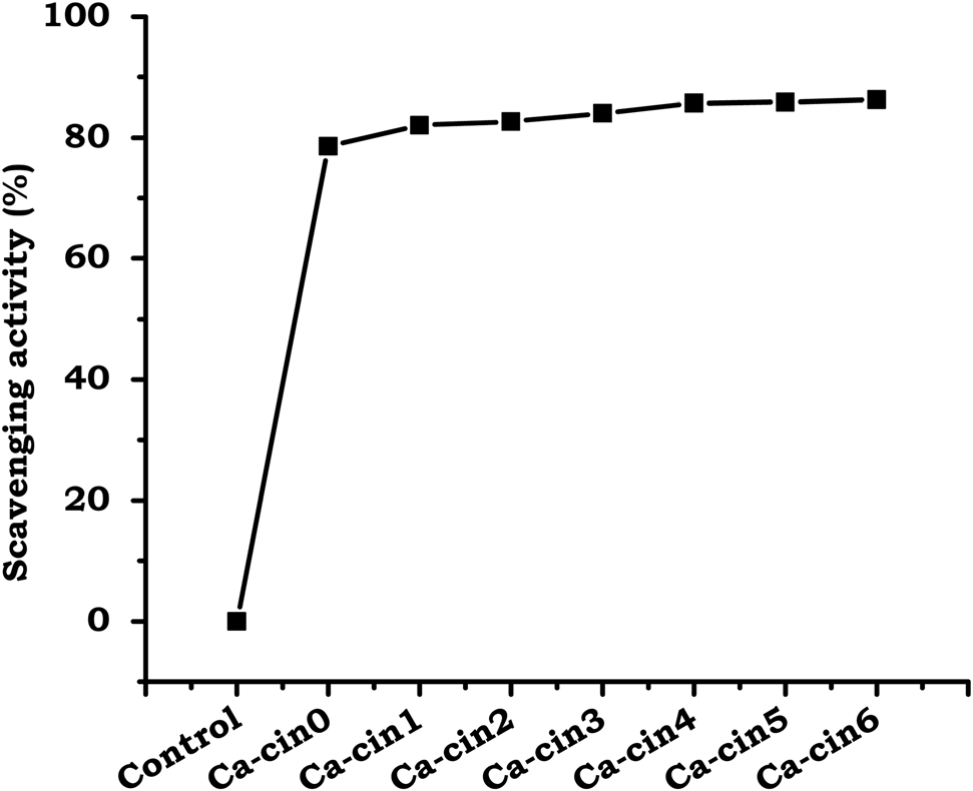
ABTS radical scavenging activity of native casein (Ca-Cin0) and cinnamaldehyde-functionalized casein derivatives (Ca-Cin1 to Ca-Cin6). Data represent the mean ± standard deviation of triplicate measurements (*n* = 3), indicating the enhanced aqueous-phase antioxidant potential of the modified samples.

The antioxidant properties of native casein and its cinnamaldehyde-modified Schiff base derivatives were assessed using the ABTS radical cation (ABTS˙^+^) decolorization assay, as illustrated in Fig. 8. The ABTS˙^+^ radical cation is characterized by a distinct bluish-green coloration and exhibits strong absorbance at 734 nm. Upon interaction with antioxidant compounds, the radical is reduced through electron or hydrogen atom donation, resulting in a measurable decrease in absorbance. This reduction in color intensity serves as an indicator of the electron-donating and radical-quenching abilities of the tested samples.^36^

As shown in Fig. 8, the cinnamaldehyde-functionalized casein derivatives exhibited a greater reduction in ABTS˙^+^ absorbance compared to native casein, reflecting improved antioxidant activity. This enhanced performance is attributed to the introduction of phenolic cinnamyl groups through Schiff base formation. Phenolic moieties are recognized for their effective electron-donating capacity, which surpasses that of the native amino groups present in unmodified casein. Their incorporation into the protein structure increases the number of active sites capable of neutralizing free radicals, thereby enhancing the overall antioxidant potential of the modified material.

These findings are consistent with previous research demonstrating the superior antioxidant properties of phenolic compounds, particularly when conjugated with biopolymers.^6^ The improved ABTS˙^+^ scavenging efficiency of cinnamyl-modified casein suggests its suitability for applications requiring oxidative protection, such as in food packaging, nutraceuticals, and biomedical formulations where oxidative stress control is essential. Notably, the ABTS scavenging activity increased markedly—up to 80%—in the Ca-Cin1 sample, indicating strong efficacy against aqueous-phase free radicals. This contrasts with the more limited activity observed in the DPPH assay, highlighting the influence of assay medium and radical type on the apparent antioxidant capacity of the modified protein.

#### 3.3.3. Bio-evaluation of casein and casein derivative

**Fig. 9:**
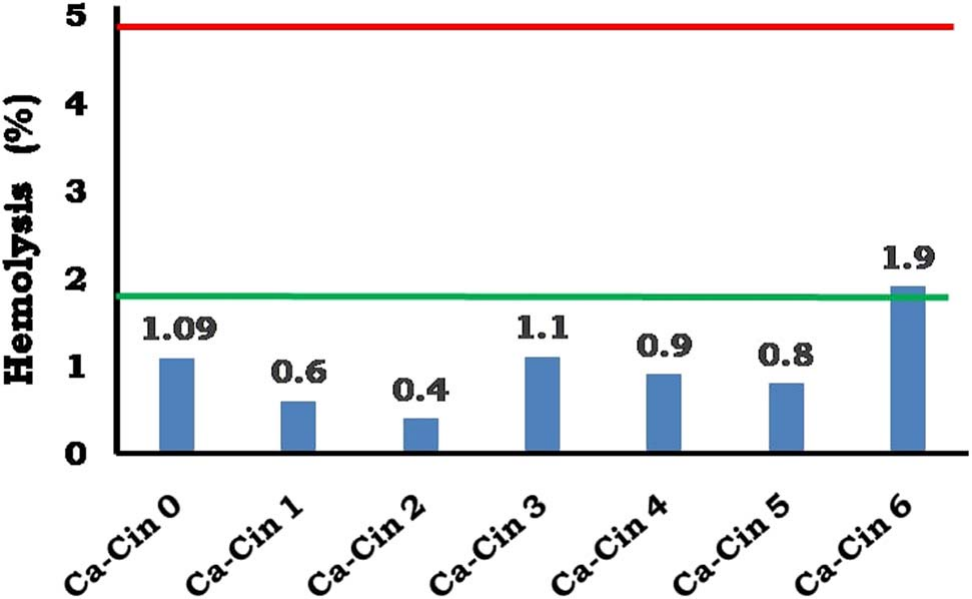
Hemolysis percent of casein and cinnamyl casein Schiff base.

The hemocompatibility of native casein and its cinnamaldehyde-functionalized Schiff base derivatives was assessed through hemolysis testing, with results presented in Fig. 9. Hemolysis assays are a standard component of biocompatibility evaluations for materials intended for blood-contacting applications, as they determine the extent of red blood cell (erythrocyte) membrane disruption upon exposure to the test material. According to ISO 10993-4, the clinical safety of such materials must be demonstrated by ensuring hemolysis remains within acceptable limits, such that potential risks do not outweigh therapeutic benefits.

The data in Fig. 9 indicate that all tested formulations—including unmodified casein (Ca-Cin0) and its cinnamyl Schiff base derivatives (Ca-Cin1 to Ca-Cin6)—exhibited hemolysis percentages below 2%, classifying them as non-hemolytic according to ASTM F 756-00. Specifically, hemolysis ranged from 1.01% for Ca-Cin0 to a maximum of 1.9% for Ca-Cin6. The marginal increase in hemolytic activity observed with higher degrees of cinnamaldehyde substitution may be attributed to changes in surface chemistry and increased hydrophobicity resulting from Schiff base formation. These modifications could potentially influence interactions with erythrocyte membranes; however, the effect remained within the non-hemolytic range.

According to ASTM F 756-00, materials are categorized based on their hemolytic index as follows: non-hemolytic (<2%), slightly hemolytic (2–5%), and hemolytic (>5%) [ASTM F 756-00, 2000]. All tested samples in this study fall well below the 2% threshold, confirming their compatibility with blood-contacting environments.

These findings are consistent with prior studies reporting that phenolic modifications, despite altering physicochemical surface properties, do not significantly impair blood compatibility when implemented at safe substitution levels.^37–39^ The cinnamaldehyde-functionalized casein derivatives demonstrated excellent hemocompatibility across all degrees of modification, suggesting their suitability for biomedical applications such as wound dressings, hemostatic agents, and drug delivery systems where minimal hemolytic response is a prerequisite.

## 4. Conclusion

In this study, we successfully synthesized cinnamaldehyde-functionalized casein Schiff bases and evaluated their potential as bioactive materials for biomedical applications. The Schiff base formation, confirmed through FTIR, TGA, and SEM analyses, demonstrated significant structural modifications and improved physicochemical properties. The modified casein derivatives exhibited enhanced antibacterial activity, particularly against Gram-positive bacteria, while showing modest effects against Gram-negative strains. Antioxidant assessments using DPPH and ABTS assays revealed that cinnamyl casein derivatives possess superior radical scavenging abilities compared to native casein, attributed to the phenolic groups introduced during functionalization. Hemocompatibility tests further confirmed the non-hemolytic nature of the modified proteins, indicating their safety for use in blood-contacting applications. These findings suggest that cinnamyl casein derivatives offer promising potential for applications in wound dressings, antimicrobial coating agents, and other biomedical fields where antioxidant and antibacterial properties are essential. Future work should focus on optimizing the degree of substitution and exploring synergistic effects with additional antimicrobial agents to enhance the bioactivity of these modified proteins further.

## Author contributions

Conceptualization, N. H., and T. M. T.; data curation, A. H., N. H., and T. M. T.; investigation, A. H., N. H., and T. M. T.; formal analysis, A. H., N. H., J. E-N., and T. M. T.; writing—original draft, A. H., N. H., J. E-N., A.S. I. A., R. M. A. S., and T. M. T. writing—review and editing, A. H., N. H., and T. M. T.; All authors have read and agreed to the published version of the manuscript.

## Conflicts of interest

The authors declare no conflict of interest.

## Data Availability

The data used in this study, including experimental results, characterization details, and analysis, can be provided upon reasonable request from the corresponding author. This ensures compliance with the journal's data sharing policies while maintaining the integrity and confidentiality of our research.
